# Consumption of whole grains and legumes modulates the genetic effect of the *APOA5* -1131C variant on changes in triglyceride and apolipoprotein A-V concentrations in patients with impaired fasting glucose or newly diagnosed type 2 diabetes

**DOI:** 10.1186/1745-6215-15-100

**Published:** 2014-04-01

**Authors:** Ryungwoo Kang, Minjoo Kim, Jey Sook Chae, Sang-Hyun Lee, Jong Ho Lee

**Affiliations:** 1 Department of Food and Nutrition, College of Human Ecology, National Leading Research Laboratory of Clinical Nutrigenetics/Nutrigenomics, Yonsei University, 50 Yonsei-ro, Seodaemun-gu, Seoul 120-749, Korea; 2 Department of Food and Nutrition, Brain Korea 21 PLUS Project, College of Human Ecology, Yonsei University, 50 Yonsei-ro, Seodaemun-gu, Seoul 120-749, Korea; 3 Yonsei University Research Institute of Science for Aging, Yonsei University, 50 Yonsei-ro, Seodaemun-gu, Seoul 120-749, Korea; 4 Department of Family Practice, National Health Insurance Corporation Ilsan Hospital, 100 ilsan-ro ilsan-donggu, Goyang 410-719, Korea

**Keywords:** *APOA5* -1131 T > C, Whole grains and legumes, Triglycerides, Apolipoprotein A-V

## Abstract

**Background:**

The apolipoprotein A5 gene (*APOA5*) -1131 T > C polymorphism is associated with mild hypertriglyceridemia in type 2 diabetic subjects, and interacts with dietary fat in the determination of triglyceride concentrations. We examined whether a substitution of whole grains and legumes for refined rice in a high carbohydrate diet (about 65% of energy derived from carbohydrate) may modify the effect of this variant on changes in apolipoprotein A-V (apoA-V) and triglyceride concentrations.

**Methods:**

We genotyped the *APOA5* -1131 T > C in individuals with impaired fasting glucose (IFG) or newly diagnosed type 2 diabetes, who were randomly assigned to either a group ingesting whole grain and legume meals daily or a control group for 12 weeks.

**Results:**

After dietary intervention, we observed significant interactions between the *APOA5* -1131 T > C polymorphism and carbohydrate sources (whole grains and legumes versus refined rice) in the determination of mean percent changes in triglyceride and apoA-V (*P* interactions <0.001 and =0.038, respectively). In the refined rice group (*n* = 93), the carriers of the risk C allele (*n* = 50) showed a greater increase in the mean percent changes of triglyceride and apoA-V than noncarriers after adjusting for HOMA-IR (*P* = 0.004 and 0.021, respectively). The whole grain and legume group (*n* = 92), however, showed a decrease in fasting glucose, HOMA-IR, and triglyceride, and an increase in apoA-V, irrespective of genotype.

**Conclusions:**

The data showed that the magnitude of the genetic effect of the *APOA5* -1131C variant on triglyceride and apoA-V levels was modulated when substituting consumption of whole grains and legumes for refined rice as a carbohydrate source in IFG or diabetic subjects.

**Trial registration:**

ClinicalTrials.gov: NCT01784952.

## Background

The role of apolipoprotein A-V (apoA-V) in the regulation of triglyceride metabolism has been demonstrated extensively in genetically modified animal models [1] and in a larger numbers of association studies [2-4]. In association studies, the most frequently analyzed variant is -1131 T > C; the C allele has been consistently associated with higher triglyceride levels. A recent Mendelian randomization experiment with the *APOA5* functional variant -1131 T > C has suggested a causal relationship between increased plasma triglyceride concentration and coronary artery disease (CAD) [5]. In support of this observation, *APOA5* -1131 T > C polymorphism has been associated with increased risk for CAD, particularly in Koreans for whom the minor allele frequency is 0.29 [2], which is much greater than the frequency reported for Caucasians (0.06) [6,7].

According to the 2010 Korean National Health and Nutrition Examination Survey (KNHANES V-1), carbohydrate-derived calories account for 65% of total caloric intake, and refined rice is the primary source of carbohydrates in middle-aged adults. This high carbohydrate intake and high prevalence of the *APOA5* -1131C allele may contribute to the relatively high mean serum triglyceride concentration (142 mg/dL) in the Korean population (KNHANES V-1). Although observational studies and intervention trials have shown that *APOA5* -1131 T > C polymorphism interacts with dietary factors (especially dietary fat) in the determination of triglyceride concentrations [8-12], the information on the relationship between the *APOA5* -1131 T > C and triglyceride induced by a high carbohydrate diet with different carbohydrate sources is scarce. Lin *et al.* have recently found elevated triglyceride levels induced by a high carbohydrate diet in healthy individuals with the *APOA5* -1131C allele [13]. Due to a clear association between diabetic dyslipidemia and *APOA5* -1131 T > C polymorphism [14] and reducing effects of a high legume low glycemic index diet on fasting triglyceride in insulin-resistant subjects [15], it was hypothesized that the substitution of whole grains and legumes for refined rice in a high carbohydrate diet could attenuate or mask the adverse effects of high carbohydrate intake in individuals with the C allele on the *APOA5* -1131 T > C polymorphism. The study focused specifically on the effects of the *APOA5* -1131 T > C polymorphism on a 12-week randomized, high carbohydrate diet intervention with different carbohydrate sources (whole grains and legumes versus refined rice), and targeted circulating levels of apoA-V and triglyceride in individuals with impaired fasting glucose (IFG) or newly diagnosed type 2 diabetes.

## Methods

### Study design and subjects

This is a randomized, open label, controlled, intervention trial to evaluate the efficacy of whole grains and legumes compared with refined rice on the genetic effect of the *APOA5* -1131C variant on changes in triglyceride and apolipoprotein A-V concentrations in patients with impaired fasting glucose or newly diagnosed type 2 diabetes.

Study subjects were selected from participants in a nutrition genomic study conducted by the National Leading Research Laboratory of Clinical Nutrigenetics/Nutrigenomics at Yonsei University. Study subjects were recruited from the Health Service Center (HSC) during a routine check-up at National Health Insurance Corporation Ilsan Hospital, Goyang, Korea (during the period January 2012 to October 2012). Based on the data screened from the HSC, subjects who were in IFG, (100 ≤ fasting glucose <126 mg/dL) and newly diagnosed type 2 diabetes (fasting glucose ≥126 mg/dL) were referred to the Department of Family Medicine or Internal Medicine. They were rechecked for their health and lipid profiles and those who satisfied the study criteria were recommended to participate in the dietary intervention program. Finally, those who consented to the program were included in this study. Exclusion criteria included: (1) current and/or past history of cardiovascular disease including angina; (2) liver or kidney dysfunction; (3) thyroid or pituitary disease; (4) unstable weight loss/gain (≥2 kg) over the previous six months; and (5) pregnancy or lactation. Subjects who were taking medication were also excluded. A total of 200 Koreans were enrolled; 15 participants subsequently discontinued the study for personal reasons, and 185 subjects (male: *n* = 39 and female: *n* = 146) completed the study. All subjects provided written informed consent before participation in this study, which was approved by the Institutional Review Board of Yonsei University.

### Dietary intervention and assessment of dietary intake/physical activity level

One week before starting the 12-week intervention, the enrolled subjects were asked to visit the center. The subjects’ usual diet information was obtained using both a 24-hour recall method and a semi-quantitative food frequency questionnaire (SQFFQ), of which the validity had been previously tested [16]. We used the former to carry out analyses and the latter to check if the data collected by 24-hour recall methods was representative of the usual dietary pattern. All the subjects were given written and verbal instructions by a registered dietitian on completion of a three-day (two weekdays and one weekend) dietary record every four weeks. Subjects were instructed to record on the sheet the amount of foods before ingestion and any remaining after ingestion by weighing the foods. All the participants were advised to continue their usual diet for one week and instructed to complete a three-day dietary record as the baseline measurement, which were then compared with the records obtained by 24-hour recall methods used on their previous visit in order to check their records.

After one week, the dietary intervention program started. Individualized and nutritionally balanced diets were planned for each subject based on their reported intakes and Korean Recommended Dietary Allowance (Korean RDA, Korean Nutrition Society, Seoul, Korea). The intervention program consisted of the replacing of refined rice intake with one third legumes, one third barleys, and one third whole grains three times per day as a high carbohydrate source, and increased vegetable intake to at least six units (30 to 70 g/unit) per day for sufficient dietary fiber intake. Participants were also instructed to record their physical activity diary for 24 hours every four weeks.

To check participants’ compliance during the entire study period, the dietitian conducted bi-weekly interviews by telephone. Participants were interviewed to discern whether they were following the program well, including dietary intake and physical activity. During the study period, all participants were also encouraged to maintain their usual lifestyle except for the instructed diet change.

Dietary energy values and nutrient content from the three-day food records were calculated using the Computer Aided Nutritional analysis program (CAN-pro 2.0, Korean Nutrition Society, Seoul, Korea). Total energy expenditure (kcal/day) was calculated from activity patterns including basal metabolic rate, physical activity for 24 hours [17], and specific dynamic action of food. Basal metabolic rate for each subject was calculated with the Harris–Benedict equation [18].

### Genotyping of *APOA5* -1131 T > C

Among the studied five common *APOA5* SNPs (-1131 T > C, -3A > G, 56C > G, 1259 T > C and IVS3 + 476G > A) in several populations [4,10,19], the -1131 T > C and the 56C > G (S19W) are considered to be functional tag SNPs [3,6,20]. However, the minor allele 19 W was found to be rare in Asian population (0.1 to 3%) [19,21-23]. Thus, only *APOA5* -1131 T > C SNP was selected as a functional SNP to investigate further. Genomic DNA was extracted from 5 mL of whole blood using a commercially available DNA isolation kit (WIZARD Genomic DNA purification kit, Promega Corp., Madison, WI, USA) according to the manufacturer’s protocol. Genotyping was performed by SNP-IT™ assays using single primer extension technology (SNPstream 25K™ System, Orchid BioSciences, NJ, United States). The colorimetric reactions were detected by an enzyme-linked immunosorbent assay (ELISA) reader, and the genotype was determined with QCReview™ software (Orchid Biosciences, Princeton, NJ, United States). Detailed methods of the clinical laboratory tests have been previously described [24].

### Anthropometric parameters and blood collection

Body weight and height were measured unclothed and without shoes in the morning. Body mass index (BMI) was calculated as body weight in kilograms divided by height in square meters (kg/m^2^). Waist circumference was measured at the umbilical level with the subjects standing after normal expiration, and the hip girth was measured at the widest part of the hip. The waist and hip ratio (WHR) was then calculated. Blood pressure (BP) was obtained from the left arm of seated patients with an automatic blood pressure monitor (TM- 2654, A&D, Tokyo, Japan) after 20 minutes of rest. After an overnight fast, venous blood specimens were collected in EDTA-(Ethylenediaminetetraacetic acid)-treated or plain tubes, and centrifuged into plasma or serum, then stored at -70°C until analysis.

### Serum lipid profile and free fatty acids

Fasting total-cholesterol and triglyceride levels were measured using commercially available kits on a Hitachi 7150 Autoanalyzer (Hitachi Ltd., Tokyo, Japan). After precipitation of apoB-(apolipoprotein B)-containing lipoproteins with dextran sulfate magnesium, HDL-(high-density lipoprotein)-cholesterol concentrations in the supernatants were enzymatically measured. LDL-(low-density lipoprotein)-cholesterol was estimated indirectly for subjects with serum triglyceride levels <400 mg/dL using the Friedwald formula: LDL - cholesterol = Total - cholesterol–(HDL - cholesterol + [Triglycerides/5]). LDL-cholesterol for subjects with serum triglyceride levels ≥400 mg/dL was measured indirectly.

### Fasting glucose, insulin concentration, and homeostasis model assessment-insulin resistance

Fasting glucose levels were measured by the glucose oxidase method with a Beckman Glucose Analyzer (Beckman Instruments, Irvine, CA, United States). Insulin levels were measured by radioimmunoassay using commercial kits from Immuno Nucleo Corporation (Stillwater, MN, United States). Insulin resistance (IR) was calculated by the homeostasis model assessment (HOMA) using the following equation: HOMA - IR = [fasting insulin(*μ*IU/mL) × fasting glucose (mmol/L)]/22.5.

### Plasma apolipoprotein A-V and serum high sensitivity C reactive protein

Plasma concentration of apolipoprotein A-V (apoA-V) was measured using an enzyme immunoassay (Human Apolipoprotein A ELISA kit, Millipore, MO, United States). The resultant color reaction was read at 450 nm using a Victor2 (Perkin Elmer life sciences, Turka, Finland). Serum high sensitivity C-reactive protein (hs-CRP) levels were measured with an Express Plus™ auto-analyzer (Chiron Diagnostics Co., Walpole, MA, United States) using a commercially available, high-sensitivity CRP-Latex(II) *X*2 kit (Seiken Laboratories Ltd., Tokyo, Japan).

### Statistical analysis

We used the SPSS version 12.0 programs for Windows (SPSS Inc., Chicago, IL, United States). Hardy–Weinberg equilibrium (HWE) analysis for *APOA5* -1131 T > C was performed by Haploview version 3.32. A paired *t*-test was used to evaluate the effects of the dietary intervention. One-way analysis of variance (ANOVA) with the Bonferroni correction or independent *t*-test was used to test the genotype effect. Pearson and partial correlation coefficients were used to determine the relationship between changes in apoA-V levels and other metabolic variables. General linear model (GLM) analysis was also used for the comparison with adjustment for confounding factors. The interaction between the -1131 T > C polymorphism and the compliance of the dietary intervention were tested in the multivariate interaction test in a regression model after controlling for potential confounders, including change in the HOMA-IR. Each variable was examined for normal distribution and skewed variables were tested after logarithmic transformation. For descriptive purposes, mean values of untransformed and unadjusted variables are presented (*P* <0.05 was considered statistically significant).

## Results and discussion

### Baseline characteristics

Baseline characteristics of participants according to the *APOA5* -1131 T > C genotype are shown in Table 1. The genotype frequencies did not deviate significantly from the Hardy-Weinberg equilibrium. The C allele frequency in subjects with IFG or new-onset type 2 diabetes was 0.314, which is slightly greater than the frequency previously reported in the healthy Korean population (0.285) [2]. Genotype frequencies were similar between men and women, and across diet groups. The *APOA5* -1131 T > C polymorphism was associated with serum levels of triglyceride (*P* < 0.001) and HDL-cholesterol (*P* = 0.013). Subjects with the C allele showed significantly higher triglyceride and lower HDL-cholesterol than those with the TT allele. There was a significant association between apoA-V levels and the -1131 T > C genotype (*P* < 0.001). Subjects with the TC and CC alleles were found to have values approximately 15% and 34% lower than those with TT, respectively. Other variables such as age, BMI, smoking and alcohol consumption, WHR, blood pressure, serum levels of total cholesterol, LDL-cholesterol, hs-CRP, glucose, insulin, free fatty acid, and HOMA-IR index were not associated with genotype at baseline (all *P* > 0.05). In order to increase statistical power for the test, we pooled carriers of the less common allele (TC + CC).

**Table 1:** **Baseline characteristic of the study participants by *APOA5 *-1131 T > C genotype**

	***APOA5 *-1131 T > C**			***P*-value**
	**TT (*n*** **= 84)**	**TC (*n*** **= 85)**	**CC (*n*** **= 16)**	
Age (year)	50.4 ± 1.08	50.1 ± 1.17	50.5 ± 1.97	0.966
Sex [*n* (%)]				0.971
F	66 (45.2)	67 (45.9)	13 (8.9)	
M	18 (46.2)	18 (46.2)	3 (7.7)	
Diet groups [*n* (%)]				0.973
Refined rice	43 (46.2)	42 (45.2)	8 (8.6)	
Whole grains and legumes	41 (44.6)	43 (46.7)	8 (8.7)	
Body mass index (kg/m^2^)	25.5 ± 0.35	25.6 ± 0.28	24.8 ± 0.46	0.604
Waist hip ratio	0.90 ± 0.01	0.89 ± 0.01	0.88 ± 0.01	0.466
Systolic BP (mmHg)	119.1 ± 1.70	122.4 ± 1.67	114.9 ± 2.90	0.135
Diastolic BP (mmHg)	77.1 ± 1.21	77.4 ± 0.99	75.1 ± 2.22	0.716
Total-cholesterol (mg/dL)^∮^	199.7 ± 3.26	98.8 ± 3.89	191.4 ± 8.59	0.583
LDL-cholesterol (mg/dL)^∮^	118.3 ± 3.19	113.9 ± 3.50	110.8 ± 8.39	0.457
hs-CRP (mg/dL)^∮^	0.70 ± 0.09	0.94 ± 0.15	0.62 ± 0.20	0.157
Glucose (mg/dL)^∮^	106.9 ± 1.72	109.5 ± 2.04	105.4 ± 4.44	0.570
Insulin (uIU/mL)^∮^	9.75 ± 0.57	9.54 ± 0.56	9.23 ± 0.82	0.978
HOMA-IR^∮^	2.56 ± 0.17	2.52 ± 0.16	2.36 ± 0.23	0.998
Free fatty acid (uEq/L)^∮^	556.0 ± 22.8	547.6 ± 20.9	514.9 ± 41.3	0.890
Triglyceride (mg/dL)^∮^	138.4 ± 6.81	180.5 ± 8.87	170.0 ± 15.3	<0.001
HDL-cholesterol (mg/dL)^∮^	53.7 ± 1.40	48.7 ± 1.23	46.6 ± 3.27	0.013
Apolipoprotein A-V (ng/mL)^∮^	233.7 ± 7.53	199.7 ± 7.47	153.6 ± 12.1	<0.001

Means ± S.E.

^∮^tested by logarithmic transformation, *P*-values derived from ANOVA with the Bonferroni method. ANOVA, Analysis of variance; BP, blood pressure; F, female; M, male; HDL, high-density lipoprotein; HOMA-IR, Homeostatic model assessment; hs-CRP, high-sensitivity C-reactive protein; LDL, low-density lipoprotein.

### Effects of the 12-week dietary intervention

At baseline and at 12 weeks, we did not find any significant difference in age, BMI, WHR, blood pressure, total cholesterol, LDL-cholesterol, and hs-CRP across the *APOA5* -1131 T > C genotype in both intervention (whole grains and legumes) and control (refined rice) groups (data not shown). Means (±SEs) of fasting glucose, insulin, HOMA-IR index, free fatty acid, triglyceride, HDL-cholesterol, and apoA-V across the *APOA5* -1131 T > C genotype by the dietary intervention and control groups at baseline and 12 weeks are shown in Table 2. The 12-week dietary intervention with whole grains and legumes significantly decreased serum levels of glucose, triglyceride, and HOMA-IR index irrespective of genotype, although the post-treatment fasting serum triglyceride level was significantly higher in C allele carriers when compared with TT allele carriers (*P* = 0.013) (Table 2). In the whole grain and legume group, C allele carriers showed a significant decrease in insulin (*P* = 0.008) and a significant increase in HDL-cholesterol (*P* = 0.020) and apoA-V (*P* = 0.005) concentrations; TT allele carriers showed a marginal decrease in insulin (*P* = 0.074) and a marginal increase in HDL-cholesterol (*P* = 0.067) and apoA-V (*P* = 0.088). In the refined rice group, C allele carriers showed an increase in triglyceride (*P* < 0.001) and apoA-V (*P* = 0.001) concentrations.

**Table 2:** **Biochemical analysis data and estimates of daily nutrient intake according to diets and *APOA5 *-1131 T > C genotype at baseline and 12 weeks**

	**Whole grains and legumes (*n*** **= 92)**				**Refined rice (*n*** **= 93)**			
	**TT (*n*** **= 41)**		**C allele (*n*** **= 51)**		**TT (*n*** **= 43)**		**C allele (*n*** **= 50)**	
	**Baseline**	**12 weeks**	**Baseline**	**12 weeks**	**Baseline**	**12 weeks**	**Baseline**	**12 weeks**
Glucose (mg/dL)^∮^	107.9 ± 2.67	97.9 ± 2.12^***^	107.7 ± 2.83	100.4 ± 2.54^***^	105.9 ± 2.21	108.8 ± 2.51^†,e^	110.1 ± 2.39	111.5 ± 2.59^f^
Insulin (uIU/mL)^∮^	9.96 ± 0.86	8.69 ± 0.65^†^	9.49 ± 0.75	8.54 ± 0.76^**^	9.54 ± 0.76	9.65 ± 0.76	9.49 ± 0.62	10.0 ± 0.77
HOMA-IR^∮^	2.61 ± 0.25	2.06 ± 0.16^**^	2.46 ± 0.22	2.08 ± 0.21^**^	2.51 ± 0.23	2.60 ± 0.24	2.52 ± 0.18	2.72 ± 0.25^f^
Free fatty acid (uEq/L)^∮^	564.1 ± 34.8	567.0 ± 29.5	548.9 ± 28.5	583.8 ± 29.9	548.0 ± 30.0	549.8 ± 37.3	535.6 ± 24.4	558.7 ± 31.5
Triglyceride (mg/dL)^∮^	139.8 ± 7.56	123.6 ± 6.85^***^	186.7 ± 11.4^a^	173.3 ± 16.8^**,b^	137.1 ± 11.3	136.3 ± 11.5	170.9 ± 10.7^c^	218.1 ± 21.7^***,d,f^
HDL-cholesterol (mg/dL)^∮^	52.3 ± 1.70	54.9 ± 1.94^†^	48.6 ± 1.37	51.2 ± 1.51^*^	55.1 ± 2.21	55.9 ± 1.98	48.2 ± 1.88^c^	49.3 ± 1.93^d^
ApoA-V (ng/dL)^∮^	225.8 ± 10.2	249.6 ± 14.0^†^	190.0 ± 9.03^a^	215.7 ± 9.70^**^	241.2 ± 11.1	239.5 ± 12.7	194.9 ± 10.2^c^	223.9 ± 9.92^**^
Total energy expenditure (kcal/d)	2050.8 ± 43.4	2046.2 ± 42.3	2047.2 ± 49.2	2037.8 ± 45.6	2018.5 ± 44.9	2009.7 ± 41.5	2066.4 ± 36.7	2055.0 ± 34.8
Estimates of daily nutrient intakes								
Energy intake (kcal/d)	2261.8 ± 47.0	2266.0 ± 45.3	2205.2 ± 52.4	2189.4 ± 45.1	2128.6 ± 45.3	2120.3 ± 43.0	2213.5 ± 39.9	2204.6 ± 38.6
Carbohydrate (%)	64.9 ± 0.93	58.9 ± 0.61^***^	65.4 ± 0.44	60.2 ± 0.59^***^	65.8 ± 0.82	65.0 ± 0.46^e^	64.6 ± 1.02	64.0 ± 0.56^f^
Protein (%)	17.1 ± 0.41	20.1 ± 0.25^***^	17.4 ± 0.29	20.4 ± 0.25^***^	7.4 ± 0.22	17.3 ± 0.20^e^	17.2 ± 0.37	16.7 ± 0.31^f^
Fat (%)	21.7 ± 0.59	24.7 ± 0.58^***^	21.6 ± 0.49	24.3 ± 0.55^***^	21.2 ± 0.43	21.8 ± 0.50^e^	21.4 ± 0.61	21.2 ± 0.46 ^f^
Crude fiber (g)^∮^	8.86 ± 0.46	11.9 ± 0.58^***^	10.7 ± 0.64	13.4 ± 0.79^***^	10.4 ± 0.65	10.6 ± 0.70	10.3 ± 0.71	10.0 ± 0.52^f^
PUFA/SFA^∮^	1.59 ± 0.14	1.82 ± 0.12^*^	1.65 ± 0.13	1.85 ± 0.11^*^	1.76 ± 0.15	1.73 ± 0.15	1.65 ± 0.14	1.49 ± 0.13^f^

Means ± S.E. HDL, high-density lipoprotein; HOMA-IR, Homeostatic model assessment, PUFA, polyunsaturated fatty acid; SFA, saturated fatty acid.

^∮^tested by logarithmic transformation.

^*a*^*P* < 0.05 comparison between TT and C allele at whole grains and legumes at baseline.

^*b*^*P* < 0.05 comparison between TT and C allele at whole grain treatment at 12 week follow-up.

^*c*^*P* < 0.05 comparison between TT and C allele at refined rice at baseline.

^*d*^*P* < 0.05 comparison between TT and C allele at refined rice at 12 week follow-up.

^*e*^*P* < 0.05 comparison between TT at whole grain and refined rice at 12 week follow-up.

^*f*^*P* < 0.05 comparison between C allele at whole grain and refined rice at 12 week follow-up.

^†^*P* < 0.1, ^*^P < 0.05, ^**^P < 0.01, ^***^P < 0.001 compared with the level at baseline in each group by paired *t*-test.

Means (±SEs) of estimates of daily nutrient intake across the *APOA5* -1131 T > C genotype by the dietary intervention and control groups at baseline and 12 weeks are shown in Table 2. In the whole grain and legume group, there were significant increases from the baseline in fiber, percent energy intake from fat and protein, and the polyunsaturated fatty acids-to-saturated fatty acids ratio.

### Interaction between *APOA5* -1131 T > C genotype and diet on 12-week changes in apoA-V and triglyceride

**Figure 1:**
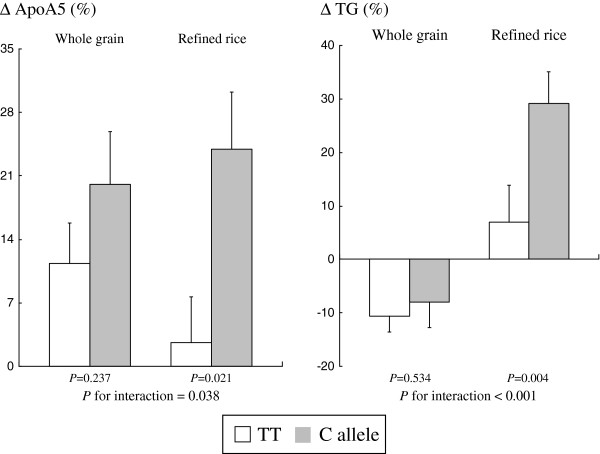
**Genotype effect of *APOA5 *-1131 T > C on mean percent changes in fasting apolipoprotein A-V and triglyceride by whole grains and legumes and refined rice groups at 12 weeks.** Means ± SE. *P*-values derived from an independent *t*-test after adjusting for change in HOMA-IR. HOMA-IR, homeostatic model assessment; TG, triglyceride.

There was no significant gene-diet interaction on serum insulin, free fatty acid, and HDL-cholesterol (data not shown). The genotype effects of *APOA5* -1131 T > C on mean (±SEs) percent changes in apoA-V and triglyceride by dietary intervention and control groups at 12 weeks are shown in Figure 1. At 12 weeks, after adjustment for age, sex, BMI, smoking and drinking, the results showed a significant interaction between the *APOA5* -1131 T > C genotype and dietary carbohydrate source (whole grains and legumes compared with refined rice) on mean percent changes in apoA-V (*P*-interaction = 0.038) and triglyceride (*P*-interaction < 0.001). In the refined rice group, subjects with the risk C allele had a greater increase in apoA-V concentration than did those with the TT allele, both before (*P* = 0.012) and after adjusting for the HOMA-IR index (*P* = 0.021), however the genetic variant was not related to changes in apoA-V in the whole grain and legume group. With respect to the triglyceride change, in the refined rice group subjects with the risk C allele had a greater increase in triglyceride concentration than did those with the TT allele, both before (*P* = 0.017) and after adjusting for the HOMA-IR index (*P* = 0.004), however the genetic variant was not related to changes in triglyceride in the whole grain and legume group.

### Relationship between changes in plasma apoA-V levels and metabolic parameter

Correlations between changes in apoA-V levels and metabolic parameters (baseline and changed levels of apoA-V, free fatty acids, triglyceride, and HDL-cholesterol) were determined after adjusting for age, sex, and BMI changes. Overall, changes in apoA-V levels were negatively correlated with baseline apoA-V (*r* = -0.345, *P* < 0.001), free fatty acid (*r* = -0.188, *P* = 0.016), and HDL-cholesterol (*r* = -0.178, *P* = 0.016), and positively correlated with changes in free fatty acid (*r* = 0.163, *P* = 0.037) and triglyceride (*r* = 0.218, *P* = 0.003). In subjects with the TT allele (*n* = 84), changes in apoA-V levels were negatively correlated with baseline apoA-V (*r* = -0.223, *P* = 0.046), free fatty acid (*r* = -0.250, *P* = 0.029), HDL-cholesterol (*r* = -0.220, *P* = 0.049), and changes in triglyceride (*r* = -0.324, *P* = 0.003) and positively correlated with changes in HDL-cholesterol (*r* = 0.296, *P* = 0.007). In C allele carriers (*n* = 101), changes in apoA-V levels were negatively correlated with baseline apoA-V (*r* = -0.408, *P* < 0.001) and positively correlated with changes in free fatty acid (*r* = 0.245, *P* = 0.025) and triglyceride (*r* = 0.458, *P* < 0.001).

In this 12-week randomized, high carbohydrate diet intervention trial (approximately 65% of energy derived from carbohydrates) with different carbohydrate sources (whole grains and legumes versus refined rice), we observed a significant interaction between the *APOA5* -1131 T > C polymorphism and carbohydrate sources in relation to mean percent changes in triglyceride and apoA-V concentrations. Compared to subjects with the TT allele, individuals with the risk C allele exhibited a greater increase in triglyceride and apoA-V responses to a control (refined-rice) diet, but had no genotype effect when assigned an intervention (whole grain and legume) diet. In previous observations, ingestion of soy protein was associated with a decrease of 10.5% in triglyceride levels [25], and replacing refined rice with legumes and increasing vegetable intake showed a 15% reduction in triglyceride levels [26]. Thus, it is acceptable that in this study, dietary intervention shows a 10% decrease in triglyceride levels in TT allele participants, while a 20% increase in C allele carriers would result from a genetic difference that exhibits higher triglyceride concentrations. These findings suggest that different carbohydrate sources in a high carbohydrate diet may modulate the genetic effect of the *APOA5* -1131C variant on changes in triglyceride and apoA-V concentrations.

Despite no significant difference in baseline triglyceride values between the individuals carrying the -1131C variant of *APOA5* gene in control and intervention groups, undergoing refined rice consumption had a higher value of triglyceride at 12 weeks. The increase in triglyceride in control subjects with the C allele is in agreement with the previous study that *APOA5* -1131C-allele carriers had higher triglyceride concentrations in response to a high-carbohydrate and low-fat diet (70 and 15% of energy derived from carbohydrate and fat, respectively) in young, healthy, Chinese adults [13]. However, the reduction of triglyceride concentrations by a 9-week low-calorie and low-fat (52 and 30% of energy derived from carbohydrate and fat, respectively) intervention was not associated with the *APOA5* -1131 T > C in overweight and obese nondiabetic Czech females [27]. These contradictory results might be partly explained by differences in calorie percent from carbohydrate (70% versus. 52%) or ethnicity (Asians versus Caucasians).

Whole grain and legume consumption is known to decrease insulin resistance or insulin demand and improve lipid profiles, including decreased triglyceride [28]. In particular, soy intake was shown to be associated with increased HDL-cholesterol and decreased triglyceride [29]. This is in agreement with our finding that -1131C carriers taking whole grains and legumes showed a significant decrease in fasting triglyceride at 12 weeks in contrast with an increase in C-allele subjects consuming refined rice. This observation of the present study could demonstrate that the magnitude of the effect of *APOA5* gene on triglyceride levels is attenuated or masked when conditions are metabolically improving, such as a decrease in glucose, insulin, and HOMA-IR index and an increase in HDL-cholesterol and apoA-V as a consequence of the replacement of refined rice with whole grain and legume. This result is consistent with the previous reports that lifestyle modification has attenuated the biological effect of genetic predisposition on metabolic profile and diabetes risk [30].

Similarly to previous observations [2,31,32], the presence of the -1131C allele was linked to lower plasma apoA-V concentrations at baseline in both control and intervention groups. After 12 weeks, however, -1131C allele carriers showed a significant increase in apoA-V concentrations, and the apoA-V levels of C allele carriers were no longer significantly different from those of TT allele carriers. This result contrasts the previous suggestion that increased triglyceride levels in individuals with the *APOA5* -1131C allele after consumption of a high-carbohydrate and low-fat diet might possibly result from reducing the translational efficiency of apoA-V in the -1131C allele [13]. This contrasting result could be partly due to increased apoA-V production resulting from high triglyceride induced by a high carbohydrate diet with refined rice. An increase in triglyceride could provide a potential explanation as to why apoA-V production in the liver is increased as a regulatory mechanism to cover the increased need of lipolysis [33]. This explanation might be supported by the earlier observations of a direct correlation of apoA-V levels to serum triglyceride in hypertriglyceridemia [34,35] and type 2 diabetes [14,36].

Although a correlation between changes in apoA-V and triglyceride was positive in -1131C allele carriers but negative in TT allele carriers, a direct correlation between changes in apoA-V and fasting free fatty acid was observed in overall subjects. Similarly, Hahne *et al.* also found an independent direct association between plasma apoA-V and free fatty acid levels in humans [37]. Due to the higher triglyceride levels in C allele carriers than TT allele carriers at baseline, it is speculated that the opposite associations between apoA-V and triglyceride according to genotype might likely be caused by the apoA-V effects on the baseline triglyceride. This observation is in line with Hyun *et al.*[24] who suggested that the negative correlation of apoA-V with triglyceride was reversed by fasting serum triglyceride partly due to the insufficient lipoprotein lipase-stimulatory activity of apoA-V in hypertriglyceridemia.

Several limitations of the study also warrant consideration. Firstly, dietary intake information was based on self-reports obtained from weighed food. However, measurement errors from self-reported dietary intake and lifestyle variables have been shown to be relatively small [38]. Secondly, because of the small sample size, the results of genetic analyses should be interpreted with caution. Finally, the euglycemic glucose clamp technique and 2-hour glucose tolerance were not performed. Despite these limitations, the results of this study indicate that IFG or diabetic subjects with the *APOA5* -1131C variant could be more susceptible than those with *APOA5* -1131TT homozygotes to the adverse effects of a high carbohydrate diet with refined rice and be more prone to having an elevation of triglyceride even with an increase in apoA-V concentration. Conversely, the magnitude of the effect of *APOA5* gene on triglyceride levels was attenuated or masked when conditions were metabolically improving in substituted consumption of whole grains and legumes for refined rice as a carbohydrate source.

## Conclusions

In IFG or diabetic individuals found to carry the *APOA5* -1131C variant, therefore, the replacement of refined rice with whole grains and legumes in a high carbohydrate diet may need to be considered to prevent diabetic hypertriglyceridemia.

## Abbreviations

APOA5: Apolipoprotein A5 gene; apoA-V: Apolipoprotein A-V; apoB: apolipoprotein B; BMI: Body mass index; BP: Blood pressure, CAD, Coronary artery disease; EDTA: Ethylenediaminetetraacetic acid; HDL: high-denstiy lipoprotein; HOMA: Homeostasis model assessment; hs-CRP: High sensitivity C reactive protein; IFG: Impaired fasting glucose; IR: Insulin resistance; LDL: low-density lipoprotein; PUFA: Polyunsaturated fatty acid; SFA: Saturated fatty acid; SNP: Single-nucleotide polymorphism; WHR: Waist and hip ratio.

## Competing interests

All authors declare that they have no competing interests.

## Authors’ contributions

RK, MK, JSC, S–HL, and JHL analyzed data, developed the study protocol and design, and read, commented on, and contributed to the submitted manuscript. JHL provided then research funding, developed the study protocol and design, and wrote the manuscript. JHL is the guarantor of this work and, as such, had full access to all the data in the study and takes responsibility for the integrity of the data and the accuracy of the data analysis. All authors read and approved the final manuscript.
